# Effects of Emotional and Sensorimotor Knowledge in Semantic Processing of Concrete and Abstract Nouns

**DOI:** 10.3389/fnhum.2012.00275

**Published:** 2012-10-08

**Authors:** P. Ian Newcombe, Cale Campbell, Paul D. Siakaluk, Penny M. Pexman

**Affiliations:** ^1^University of Northern British ColumbiaPrince George, BC, Canada; ^2^University of CalgaryCalgary, AB, Canada

**Keywords:** emotional experience, imageability, body-object interaction, semantic richness, grounded cognition

## Abstract

There is much empirical evidence that words’ relative imageability and body-object interaction (BOI) facilitate lexical processing for concrete nouns (e.g., Bennett et al., 2011). These findings are consistent with a grounded cognition framework (e.g., Barsalou, 2008), in which sensorimotor knowledge is integral to lexical processing. In the present study, we examined whether lexical processing is also sensitive to the dimension of emotional experience (i.e., the ease with which words evoke emotional experience), which is also derived from a grounded cognition framework. We examined the effects of emotional experience, imageability, and BOI in semantic categorization for concrete and abstract nouns. Our results indicate that for concrete nouns, emotional experience was associated with less accurate categorization, whereas imageability and BOI were associated with faster and more accurate categorization. For abstract nouns, emotional experience was associated with faster and more accurate categorization, whereas BOI was associated with slower and less accurate categorization. This pattern of results was observed even with many other lexical and semantic dimensions statistically controlled. These findings are consistent with Vigliocco et al.’s (2009) theory of semantic representation, which states that emotional knowledge underlies meanings for abstract concepts, whereas sensorimotor knowledge underlies meanings for concrete concepts.

## Introduction

Classical theories of cognition hold that perception and cognition have distinct representational formats, such that perceptual representations are modal, whereas conceptual representations are amodal (Fodor, 1983; Pylyshyn, 1984). More recently, a growing number of cognitive scientists have proposed an alternative theoretical perspective, called embodied or grounded cognition, in which modal representations underlie both perceptual and conceptual knowledge (e.g., Pecher and Zwaan, 2005). One well-developed and influential grounded cognition framework is perceptual symbol systems (PSS: Barsalou, 1999). According to PSS, conceptual knowledge is largely acquired through bodily interaction with the environment and is inherently multimodal, such that different aspects of conceptual knowledge are stored in different neural systems dedicated to sensorimotor processing (e.g., sensory knowledge is stored in neural systems dedicated to sensory processing, whereas motor knowledge is stored in neural systems dedicated to motor processing). Conceptual processing occurs via simulation, or the partial reenactment of the various neural states that were involved during bodily interaction with the environment. More recently, Barsalou (2003, 2008, 2009) has emphasized that an important aspect of the acquisition and subsequent simulation of conceptual knowledge is that it does not occur in a contextual vacuum. That is, “(a)t any given moment in perception, people perceive the immediate space around them, including agents, objects, and events present” (Barsalou, 2009, p. 1283). Barsalou (2003) referred to the fact that conceptual knowledge is influenced by environmental context as *situated conceptualization*.

The PSS framework has recently been extended in empirical efforts investigating whether sensorimotor knowledge influences lexical processing. Several studies have examined the influence of knowledge gained through sensory experience, as measured by imageability (i.e., how easily words evoke mental images), in lexical processing. Imageability has been shown to facilitate responding in lexical decision, word naming, picture naming, progressive demasking, and semantic categorization tasks (Balota et al., 2004; Bennett et al., 2011; Yap et al., 2012). Additional studies have examined the influence of knowledge gained through motor experience in lexical processing, as indexed by a dimension known as body-object interaction (BOI), which measures perceptions of the ease with which a human body can physically interact with a word’s referent. BOI has been shown to facilitate responding in lexical decision, phonological lexical decision, word naming, picture naming, semantic categorization, and sentence processing tasks (Siakaluk et al., 2008a,b; Tillotson et al., 2008; Bennett et al., 2011; Wellsby et al., 2011; Hansen et al., 2012; Phillips et al., 2012; Tousignant and Pexman, 2012; Yap et al., 2012). Further, in an fMRI study involving the semantic categorization task (SCT), processing of high BOI words was associated with greater activation in the left inferior parietal lobule (supramarginal gyrus, BA 40), a sensory association area involved in kinesthetic memory (Hargreaves et al., 2012). This finding suggests that knowledge about the relative availability of motor experience with words’ referents is activated during visual word recognition. The facilitatory effects of imageability and BOI arise, according to the PSS framework, because easily imageable words and high BOI words refer to concrete concepts that occur in environmental contexts that allow development of relatively rich stores of sensory knowledge and motor knowledge, and elicit richer sensory simulations and motor simulations. That is, by virtue of their associated sensory knowledge and motor knowledge, easily imageable words and high BOI words enjoy *semantic richness*.

The semantic feedback activation framework describes in more explicit detail how semantic richness effects, such as the facilitatory effects of imageability and BOI, arise within the visual word recognition system (Hino and Lupker, 1996; Pexman et al., 2002). According to this framework, the visual word recognition system is comprised of separate but interconnected sets of units dedicated to processing orthographic, phonological, and semantic information. In tasks in which responses are based primarily on orthographic processing (e.g., lexical decision), semantically richer words (i.e., easily imageable words and high BOI words) generate greater levels of semantic activation (i.e., richer sensory simulations and richer motor simulations) within semantic units, which leads to greater semantic feedback activation to orthographic units, thus leading to faster responding for these words. The same explanation holds in tasks in which responses are based primarily on phonological processing (e.g., word naming), except that the relevant semantic feedback activation is that which influences phonological units. In tasks in which responses are based primarily on semantic processing (e.g., semantic categorization), greater levels of semantic activation (i.e., richer sensory simulations and richer motor simulations) generated by semantically richer words (i.e., easily imageable words and high BOI words) leads to faster settling of their associated semantic representations within semantic units. Notably, while many studies have reported facilitatory effects of various semantic richness dimensions in SCTs, it has also been established that the nature of these effects will depend on the particular decision category involved, and how the richness dimension is relevant to that category (e.g., Pexman et al., 2003; Tousignant and Pexman, 2012).

### Grounding lexical semantics beyond sensorimotor knowledge

Recently, it has been noted that researchers with a grounded cognition perspective have focused primarily on the sensorimotor aspects of cognition, and have largely ignored other types of knowledge that are relevant to this perspective, such as the emotional aspects of cognition (Vigliocco et al., 2009; Parisi, 2011). This is an important consideration, because there is accumulating evidence that emotional knowledge plays a number of roles in cognition more generally (Dolan, 2002; Vigliocco et al., 2009), and in conceptual processing more particularly (Niedenthal et al., 2005a,b, 2009; Wilson-Mendenhall et al., 2011).

Wilson-Mendenhall et al. (2011) extended the PSS framework to account for how emotional concepts may be acquired through bodily experience via situated conceptualization. That is, they suggested that emotional knowledge is acquired and simulated in the same manner in which sensorimotor knowledge is acquired and simulated. They stated, “Like all concepts, emotion concepts originate and operate in the context of continuous situated activity, with situations typically including a physical setting, agents, objects, and actions in the world, interoceptive sensations from the body, and mentalizing related to prospective and retrospective thought” (p. 1108). Thus, although emotional knowledge can be considered more abstract in nature than sensorimotor knowledge, both types of knowledge are nonetheless acquired and simulated through the same mechanisms. More specifically, emotional knowledge is stored in the neural systems dedicated to emotional processing, and conceptual processing of emotional knowledge occurs through simulation, or the partial reenactment of the various neural states that were involved during bodily interaction with the environment.

Vigliocco et al. (2009) developed a similar framework, and argued that emotional knowledge is grounded in bodily experience and is integral to conceptual processing. According to Vigliocco et al.’s (2009) framework of semantic representation, there are two general classes of knowledge that humans are able to acquire and use in conceptual processing. One type is what they refer to as *experiential* knowledge and the other type is what they refer to as *linguistic* knowledge. They characterize experiential knowledge as being derived not only from sensory and motor experience with the external environment, but also from affective or emotional experience with the internal environment of one’s own body (e.g., the experience of moods or feelings) in conjunction with external events. They characterize linguistic knowledge as lexical co-occurrence information (e.g., the Topics model, Griffiths et al., 2007; the LSA model, Landauer and Dumais, 1997; the HAL model, Lund and Burgess, 1996), and syntactic information^1^. Importantly, they further propose that sensorimotor knowledge is more salient to the acquisition and use of concrete concepts than abstract concepts, whereas emotional knowledge and linguistic knowledge types are more salient to the acquisition and use of abstract concepts than concrete concepts. In other words, sensorimotor knowledge can be thought of as diagnostic of concrete concepts, whereas emotional knowledge and linguistic knowledge can be thought of as diagnostic of abstract concepts.

In support of the idea that emotional knowledge is integral to the processing of abstract concepts in lexical processing, Kousta et al. (2009) reported faster lexical decision latencies for negative words and for positive words than for neutral words (there was no difference in latencies between the negative words and the positive words). Importantly, these facilitatory effects of emotion were independent of any influence of concreteness or imageability (all three sets of words were matched on these two dimensions). In addition, Kousta et al. (2011) reported an intriguing result they called the *abstractness effect*. When imageability and context availability were statistically controlled, Kousta et al. (2011) reported that lexical decision latencies were faster for abstract words than for concrete words, in contrast to the typical finding whereby latencies are faster for concrete words than for abstract words. They attributed this reversal to the facilitatory influence of emotional content for abstract words. Thus, both Vigliocco et al.’s (2009) and Wilson-Mendenhall et al.’s (2011) frameworks conceive emotional knowledge as situationally and experientially derived, and we use those characterizations to frame predictions in the current study.

### The present study

The primary purpose of the present study was to examine the relative contributions of emotional, sensory, and motor knowledge to semantic processing for concrete and abstract nouns. In doing so, our objective was to test Vigliocco et al.’s (2009) claims that emotional knowledge underlies the meanings of abstract nouns, whereas sensory and motor knowledge underlies the meanings of concrete nouns. More specifically, we assessed emotional knowledge using a new dimension we call *emotional experience*, which was designed to capture the relative ease with which words elicit or evoke emotional experience. We assessed sensory knowledge using the dimension of imageability, and we assessed motor knowledge using the dimension of BOI. We examined the behavioral effects of the above three dimensions in semantic categorization for nouns referring to concrete concepts and for nouns referring to abstract concepts. We characterized emotional experience as a unitary dimension to make it analogous to imageability and BOI, and thus to facilitate comparisons between these three dimensions of experiential knowledge.

An example may help elucidate how different types of experiential knowledge may underlie the development and activation of concrete and abstract conceptual knowledge. Imagine a situation in which you are very thirsty and have been looking for some time for something to drink. When you finally see a fountain, you run to it, take a good drink of water, and feel relieved. This situation involves the concrete concept of “fountain” and the abstract concept of “relief.” The concrete noun *fountain* can be considered easily imageable, because it refers to things that can be easily perceived by the senses (e.g., sight, touch), and high on the BOI dimension, because it also refers to things that can be easily physically interacted with (e.g., turning the knob to start the flow of water, holding the fountain for balance, bending down to drink the water). We suggest that *fountain* can be considered low on the emotional experience dimension, because it is unlikely that it refers to things that are reliably associated with relatively robust emotional experiences (e.g., when one sees fountains, are they always associated with emotional experiences as they are with visual experiences and motor experiences?). Thus, what people know about the concrete concept “fountain” will be derived primarily from sensory experience and motor experience, and perhaps only secondarily from emotional experience. Conversely, the abstract noun *relief* can be considered high on the emotional experience dimension, because it refers to the alleviation or deliverance from distress (e.g., drinking water to quench thirst). *Relief* can also be considered not easily imageable, because it cannot be easily perceived by the senses, and low on the BOI dimension, because it cannot be easily physically interacted with (if at all). Thus, what people know about the abstract concept “relief” will be derived primarily from emotional experience, and perhaps only secondarily (if at all) from sensory experience and motor experience.

According to Vigliocco et al.’s (2009) framework of semantic representation, there is a “statistical preponderance for sensory-motor information to underlie concrete word meanings and a preponderance for affective (i.e., emotional)…information to underlie abstract word meanings” (p. 223). The implications of these claims are that sensory knowledge and motor knowledge should be especially salient to the processing of concrete nouns, whereas emotional knowledge should be especially salient to the processing of abstract nouns. We selected our stimuli to be either concrete or abstract (described below), and presented these stimuli in two separate tasks. In the concrete SCT, the decision criterion was to decide if the stimuli referred to concrete nouns, whereas in the abstract SCT, the decision criterion was to decide if the stimuli referred to abstract nouns. We propose that there are two benefits in using this experimental design. First, the decision criteria allowed for a relatively more pure assessment of the effects of the three dimensions of experiential knowledge than would a task such as lexical decision. This is because each SCT directly emphasizes processing of the relevant semantic characteristic under examination, namely concreteness in the concrete SCT and abstractness in the abstract SCT, rather than requiring a more peripheral decision to be made in lexical decision (e.g., is the item a word). Second, the decision criteria allowed a more refined analysis of the individual effects of each of the three dimensions of experiential knowledge in each SCT. That is, according to the semantic feedback activation framework, the nature of the effects of any given semantic richness dimension in the SCT will depend on whether the dimension is congruent with the decision category (Pexman et al., 2003; Tousignant and Pexman, 2012).

As such, we made the following prediction regarding the effects of imageability and BOI on the processing of concrete nouns in the concrete SCT: both dimensions should facilitate categorization, such that higher ratings on these two dimensions should be associated with faster and more accurate categorizations, because these two dimensions are diagnostic of concrete concepts, and thus are congruent with the decision criterion of “is the word concrete?”. We made the following two more speculative predictions regarding the effects of emotional experience on the processing of concrete concepts in the concrete SCT. One possibility, derived from the semantic feedback activation framework of visual word recognition (Hino and Lupker, 1996; Pexman et al., 2002), is that because emotional experience is diagnostic of abstract concepts, it thus may inhibit categorization in the concrete SCT, because this dimension is not congruent with the decision criterion of “is the word concrete?”. An alternative possibility is that no effects of this dimension will be observed, and this may arise either because the effects of emotional experience may be too subtle to detect using the type of experimental design employed in the present study (although they may be detectable using other experimental tasks), or they play little or no role in the processing of concrete concepts (admittedly, this is a very strong interpretation of Vigliocco et al.’s (2009), framework of semantic representation), or for some other reason.

We made the following prediction regarding the effects of emotional experience on the processing of abstract nouns in the abstract SCT: emotional experience should facilitate categorization, such that higher ratings on this dimension should be associated with faster and more accurate categorizations, because emotional experience is diagnostic of abstract concepts, and thus is congruent with the decision criterion of “is the word abstract?”. We made the following two more speculative predictions regarding the effects of imageability and BOI on the processing of abstract concepts in the abstract SCT. One possibility, derived from the semantic feedback activation framework of visual word recognition (Hino and Lupker, 1996; Pexman et al., 2002), is that because these two dimensions are diagnostic of concrete concepts, they thus may inhibit categorization in the abstract SCT, because these dimensions are not congruent with the decision criterion of “is the word abstract?”. An alternative possibility is that no effects of these two dimensions will be observed, and this may arise for the same reasons outlined in the paragraph above (i.e., the effects of imageability and BOI may be too subtle to detect using the type of experimental design employed in the present study, or they play little or no role in the processing of abstract concepts, or for some other reason).

## Materials and Methods

### Participants

Two separate groups of 30 undergraduate students from the University of Northern British Columbia participated for bonus course credit: one group participated in the concrete SCT and the other group participated in the abstract SCT. All were native English speakers and reported normal or corrected-to-normal vision.

### Stimuli

Two hundred concrete nouns and 200 abstract nouns were selected from the Toronto Word Pool (Friendly et al., 1982) or the Paivio et al. (1968) word banks. The concrete and abstract nouns are listed in Concrete Nouns Used in the Experiments and Abstract Nouns Used in the Experiments in Appendix, respectively. Concrete nouns had concreteness and imageability ratings of 5.0 or higher, whereas abstract nouns had concreteness and imageability ratings of 3.9 or less. The concrete nouns and the abstract nouns were matched pairwise on print length. Values were obtained for the following control variables: HAL log-frequency, Levenshtein orthographic distance, number of letters, phonemes, syllables, and morphemes (all taken from Balota et al., 2007), age of acquisition (AoA, taken from Kuperman et al., 2012), concreteness^2^, to control for typicality effects (as noted, taken from either Friendly et al. or Paivio et al.), number of senses (retrieved from the www.wordsmyth.net), and the inverse of the number of neighbor words (plus 1) within the neighborhood threshold (NCOUNT-INV)^3^ (Shaoul and Westbury, 2010a,b). The semantic richness variables of interest for the present study included: emotional experience, imageability, and BOI. Emotional experience ratings and BOI ratings were collected from two separate groups of 30 undergraduate students from the University of Calgary. The instructions used for the emotional experience ratings were derived for the present study and are given in Written Instructions Used for the Emotional Experience Rating Task in Appendix. The instructions used for the BOI ratings were the same as those used by Tillotson et al. (2008).

### Apparatus and procedure

The 200 concrete nouns and the 200 abstract nouns were presented in both tasks. For the concrete SCT, participants were instructed to decide only whether each word referred to a concrete noun, and to respond by pressing the “?” key on the computer keyboard if the word did refer to a concrete noun and to not press any key if the word did not refer to a concrete noun (i.e., participants were instructed to respond only to the concrete nouns). For the abstract SCT, participants were instructed to decide only whether each word referred to an abstract noun, and to respond by pressing the “?” key if the word did refer to an abstract noun and to not press any key if the word did not refer to an abstract noun (i.e., participants were instructed to respond only to the abstract nouns). Participants were instructed to make their responses as quickly and as accurately as possible, and were told that the stimuli for which they did not make a response would be automatically replaced by the next stimulus item after 2500 ms. The stimuli were presented in the center of a color VGA monitor driven by a Pentium-class microcomputer running DirectRT software^4^. A trial was initiated by a fixation marker that appeared at the center of the computer display for 1000 ms and was then replaced by a stimulus item. The intertrial interval was 2000 ms. Stimulus order was randomized separately for each participant. Following every 100 trials, participants had an opportunity to take a break, and continued when ready by pressing the spacebar. Before beginning either task, participants were given practice trials consisting of 10 concrete nouns and 10 abstract nouns.

### Data analysis

The data from both tasks were first analyzed jointly to test for interaction effects between task (i.e., concrete, abstract) and each of emotional experience, imageability, and BOI. The following variables were entered in the first step of a hierarchical multiple regression analysis: HAL log-frequency, AoA, Levenshtein orthographic distance, number of phonemes, syllables, and morphemes, concreteness, number of senses, NCOUNT-INV, type of task (dummy coded; “1” for concrete nouns, “2” for abstract nouns), and emotional experience, imageability, and BOI^5^. The final three variables were centered prior to inclusion in the analysis (Keith, 2006). The following interaction variables were entered in the second step: task by emotional experience, task by imageability, and task by BOI. These three interaction terms were constructed by creating cross-product terms through the multiplication of the task variable with the appropriate centered semantic richness variable (Keith, 2006). To follow up any significant interactions, we then examined the effects of emotional experience, imageability, and BOI in each data set separately in two additional hierarchical multiple regression analyses. In these follow up analyses, the following variables were entered in the first step: HAL log-frequency, AoA, Levenshtein orthographic distance, number of phonemes, syllables, and morphemes, concreteness, number of senses, and NCOUNT-INV. Emotional experience, imageability, and BOI were then entered in the second step. We used hierarchical multiple regression because it provided two important pieces of information, namely, the change in *R*^2^ when the three dimensions of experiential knowledge were added to the analyses (after a number of control variables were already entered), and whether each of the three dimensions of experiential knowledge accounted for a significant amount of unique variability in semantic processing.

## Results

There were 10 concrete nouns (from the concrete SCT data) and 10 abstract nouns (from the abstract SCT data) that had error rates greater than 30%. In addition, the abstract nouns *justice* and *moment* were used as examples in the emotional experience ratings instructions. Therefore, in the omnibus categorization latency and categorization error analyses, the 10 concrete nouns and the 10 abstract nouns with high error rates, along with the two abstract nouns used in the emotional experience ratings instructions, were removed. Further, in the follow up analyses of the concrete SCT data, only the 10 concrete nouns had to be removed, whereas in the follow up analyses of the abstract SCT data, only the 10 abstract nouns and the two abstract nouns used in the emotional experience ratings instructions had to be removed. The items that were removed from the analyses are indicated with * in Concrete Nouns Used in the Experiments and Abstract Nouns Used in the Experiments in Appendix. Outliers were identified in the following manner. First, categorization latencies faster than 250 ms or slower than 2000 ms were considered outliers. Second, for each participant, categorization latencies greater than 2.5 SDs from the mean were considered outliers. Using this procedure, a total of 151 observations (2.6% of the data) were removed from the concrete SCT data set, and a total of 178 observations (3.2% of the data) were removed from the abstract SCT data set. The raw categorization latencies were *z* score transformed before analysis.

### Omnibus analysis

Means and SDs for the predictor variables for the concrete nouns and the abstract nouns are shown in Table 1 (note that we included the uncentered means for the emotional experience, imageability, and BOI variables, and that the SDs are identical whether using the uncentered or centered means). Zero-order correlations between the criterion variables and the predictor variables for the concrete SCT are presented in Table 2, and zero-order correlations between the criterion variables and the predictor variables for the abstract SCT are presented in Table 3. For the regression analyses, the critical results are those for the three interaction tests at step 2, and thus only these results are shown in Table 4. For both criterion variables (categorization latencies, categorization errors), there was a significant change in *R*^2^ when the three interaction terms were added to the analyses, and importantly, each of the three interaction tests were significant. Interestingly, and consistent with Vigliocco et al.’s (2009) framework of semantic representation, the effects of the imageability and BOI dimensions were in the same direction, whereas the effects of the emotional experience dimension was in the opposite direction. These significant interactions mean that the regression lines for each dimension of experiential knowledge are not parallel (i.e., they have significantly different slopes) in the two SCTs. To better understand the precise nature of the effects of emotional experience, imageability, and BOI for each SCT, we conducted follow up hierarchical multiple regression analyses separately for each data set.

**Table 1:** **Descriptive statistics and behavioral data for the 190 concrete nouns (from the concrete SCT) and the 188 abstract nouns (from the abstract SCT)**.

Variable	Concrete nouns		Abstract nouns	
	*M*	SD	*M*	SD
Log-frequency (HAL)	8.53	1.79	8.88	1.82
Age of acquisition	6.70	1.98	9.65	2.31
Levenshtein orthographic distance	2.60	0.92	2.51	0.64
Letters	7.14	1.75	7.22	1.73
Phonemes	5.67	1.66	6.15	1.65
Syllables	2.24	0.65	2.47	0.84
Morphemes	1.39	0.61	1.70	0.68
Concreteness	6.16	0.52	2.57	0.69
Senses	2.62	1.59	3.21	1.63
NCOUNT-INV	0.23	0.41	0.22	0.41
Emotional experience	2.18	0.77	3.39	1.10
Imageability	5.80	0.63	3.00	0.57
Body-object interaction	4.89	0.93	2.00	0.33
Raw categorization latencies	783.96	115.30	918.32	93.64
Categorization errors	4.09	6.91	5.44	5.94

*NCOUNT-INV, inverse of number of word neighbors plus 1*.

**Table 2:** **Zero-order correlations between the criterion variables and the predictor variables for the concrete SCT**.

Measure	1	2	3	4	5	6	7	8	9	10	11	12	13	14	15
1. CL	–														
2. Errors	0.63**	–													
3. Freq	−0.09	0.04	–												
4. AoA	0.59**	0.32**	−0.31**	–											
5. LOD	0.06	−0.10	−0.63**	0.30**	–										
6. Letters	0.13	−0.01	−0.58**	0.27**	0.89**	–									
7. Phon	0.15*	−0.01	−0.50**	0.29**	0.79**	0.83**	–								
8. Syll	0.10	0.03	−0.42**	0.19**	0.70**	0.72**	0.78**	–							
9. Morph	0.04	−0.06	−0.37**	0.07	0.38**	0.48**	0.39**	0.30**	–						
10. Conc	−0.58**	−0.50**	0.14	−0.43**	−0.35**	−0.39**	−0.46**	−0.37**	−0.24**	–					
11. Senses	0.10	0.13	0.48**	−0.16*	−0.33**	−0.32**	−0.34**	−0.29**	−0.20**	−0.02	–				
12. INV + 1	−0.05	−0.11	−0.70**	0.10	0.59**	0.55**	0.48**	0.38**	0.35**	−0.17*	−0.36**	–			
13. EE	0.04	0.20**	0.39**	−0.09	−0.19**	−0.20**	−0.15*	−0.15*	−0.20**	−0.09	0.17*	−0.30**	–		
14. Image	−0.61**	−0.49**	0.15*	−0.61**	−0.19**	−0.22**	−0.24**	−0.18*	−0.08	0.66**	0.04	−0.08	0.05	–	
15. BOI	−0.62**	−0.64**	−0.02	−0.37**	0.07	0.02	0.00	0.04	0.06	0.49**	−0.12	0.14	0.00	0.41**	–

*p < 0.05, **p < 0.01.

*CL, categorization latency; Freq, HAL log-frequency; AoA, age of acquisition; LOD, Levenshtein orthographic distance; Letters, number of letters; Phon, number of phonemes; Syll, number of syllables; Morph, number of morphemes; Conc, concreteness; Senses, number of senses; INV + 1, inverse of number of word neighbors plus 1; EE, emotional experience; Image, imageability; BOI, body-object interaction*.

**Table 3:** **Zero-order correlations between the criterion variables and the predictor variables for the abstract SCT**.

Measure	1	2	3	4	5	6	7	8	9	10	11	12	13	14	15
1. CL	–														
2. Errors	0.42**	–													
3. Freq	−0.26**	0.02	–												
4. AoA	0.24**	0.00	−0.63**	–											
5. LOD	0.12	−0.07	−0.39**	0.37**	–										
6. Letters	0.12	−0.11	−0.39**	0.39**	0.79**	–									
7. Phon	0.25**	−0.01	−0.42**	0.47**	0.71**	0.84**	–								
8. Syll	0.22**	−0.04	−0.41**	0.49**	0.63**	0.70**	0.75**	–							
9. Morph	0.11	0.01	−0.32**	0.36**	0.49**	0.69**	0.62**	0.64**	–						
10. Conc	0.24**	0.39**	0.18*	−0.12	−0.20**	−0.16*	−0.12	−0.18*	−0.11	–					
11. Senses	−0.21**	−0.09	0.47**	−0.40**	−0.27**	−0.27**	−0.30**	−0.20**	−0.19**	0.04	–				
12. INV + 1	0.22**	0.00	−0.71**	0.43**	0.24**	0.17*	0.22**	0.25**	0.20**	−0.20**	−0.37**	–			
13. EE	−0.45**	−0.32**	0.09	−0.28**	0.04	0.01	−0.05	−0.05	−0.12	−0.26**	0.16*	−0.14	–		
14. Image	−0.12	−0.03	0.01	−0.24**	0.08	0.09	0.03	0.00	−0.05	0.09	0.13	0.01	0.49**	–	
15. BOI	0.09	0.28**	0.11	−0.26**	−0.01	−0.03	−0.01	−0.02	−0.10	0.23**	0.09	−0.11	0.30**	0.43**	–

*p < 0.05, **p < 0.01.

*CL, categorization latency; Freq, HAL log-frequency; AoA, age of acquisition; LOD, Levenshtein orthographic distance; Letters, number of letters; Phon, number of phonemes; Syll, number of syllables; Morph, number of morphemes; Conc, concreteness; Senses, number of senses; INV + 1, inverse of number of word neighbors plus 1; EE, emotional experience; Image, imageability; BOI, body-object interaction*.

**Table 4:** **Results of interaction tests in the omnibus analyses**.

Variable	*B*	SEB	β	sr	Δ*R^2^*	*R^2^*
**CATEGORIZATION LATENCY**						
Step 1 (control variables)						0.35***
Step 2					0.14***	0.49***
Task × EE	−0.16	0.03	−0.81	−0.20***		
Task × imageability	0.25	0.05	0.66	0.18***		
Task × BOI	0.39	0.07	0.93	0.22***		
**CATEGORIZATION ERROR**						
Step 1 (control variables)						0.22***
Step 2					0.20***	0.42***
Task × EE	−4.05	0.64	−1.03	−0.25***		
Task × imageability	3.98	1.06	0.56	0.15***		
Task × BOI	10.46	1.35	1.30	0.31***		

*p < 0.05, **p < 0.01, ***p < 0.001.

*EE, emotional experience; BOI, body-object interaction*.

### Concrete SCT

The hierarchical multiple regression results are shown in Table 5. (For both SCTs, the associated beta-weights and semi-partial correlations for the predictor variables are given only for the step at which they entered the multiple regression equation.) There are two important results that should be highlighted. First, at step 1 of the analyses, concreteness had significant negative semi-partial correlations for both categorization latencies and categorization errors. That is, concreteness exerted a facilitatory effect, such that higher concreteness ratings (i.e., higher typicality ratings for the “concrete” category, see footnote 2) were associated with faster and more accurate categorizations (which is exactly what would be expected from “concrete” typicality ratings). The within-category variability accounted for by concreteness (or typicality) allowed for a more stringent test of the effects of the three dimensions of experiential knowledge at step 2 of the analyses. Second, and importantly, as can be seen in Table 5, for both criterion variables, there was a significant change in *R*^2^ when the three dimensions of experiential knowledge were added to the analyses.

**Table 5:** **Results of hierarchical multiple regression analyses for the concrete SCT**.

Variable	*B*	*SEB*	β	*sr*	Δ*R^2^*	*R^2^*
**CATEGORIZATION LATENCY**						
Step 1						0.55***
Freq	−0.04	0.02	−0.20	−0.12*		
AoA	0.08	0.01	0.43	0.35***		
LOD	−0.11	0.04	−0.27	−0.14**		
Phonemes	0.00	0.02	0.00	0.00		
Syllables	0.03	0.05	0.04	0.03		
Morphemes	−0.02	0.04	−0.02	−0.02		
Concreteness	−0.35	0.05	−0.47	−0.37***		
Senses	0.03	0.01	0.13	0.11*		
NCOUNT−INV	−0.11	0.07	−0.12	−0.08		
Step 2					0.07***	0.62***
EE	0.03	0.03	0.06	0.06		
Imageability	−0.12	0.04	−0.19	−0.12**		
BOI	−0.13	0.03	−0.31	−0.24***		
**CATEGORIZATION ERROR**						
Step 1						0.39***
Freq	−0.66	0.38	−0.17	−0.10		
AoA	0.57	0.25	0.16	0.13*		
LOD	−2.43	0.83	−0.32	−0.17**		
Phonemes	−0.75	0.49	−0.18	−0.09		
Syllables	1.67	1.03	0.16	0.09		
Morphemes	−1.00	0.75	−0.09	−0.08		
Concreteness	−7.62	0.99	−0.57	−0.45***		
Senses	0.25	0.30	0.06	0.05		
NCOUNT-INV	−1.33	1.47	−0.08	−0.05		
Step 2					0.16***	0.55***
EE	1.85	0.51	0.21	0.18***		
Imageability	−2.75	0.86	−0.25	−0.16**		
BOI	−3.41	0.49	−0.46	−0.35***		

*p < 0.05, **p < 0.01, ***p < 0.001.

*Freq, HAL log-frequency; AoA, age of acquisition; LOD, Levenshtein orthographic distance; NCOUNT-INV, inverse of number of word neighbors plus 1; EE, emotional experience; BOI, body-object interaction*.

Recall that according to Vigliocco et al.’s (2009) framework of semantic representation, sensorimotor knowledge is diagnostic of concrete concepts. We therefore had predicted that imageability and BOI should exert facilitatory effects in the concrete SCT. These predictions were supported for both the categorization latency and categorization error data, such that higher imageability ratings and higher BOI ratings were associated with faster and more accurate categorizations. Our predictions regarding the effects of emotional experience were more speculative. One possibility we suggested was that because emotional experience is diagnostic of abstract concepts, it may inhibit categorization, because this dimension is not congruent with the decision criterion of “is the word concrete?”. A second possibility we suggested was that this dimension may exert no effects (for the potential reasons outlined above). The data provided mixed support for the two predictions. There was an inhibitory effect of emotional experience on categorization errors, such that higher emotional experience ratings were associated with less accurate categorizations, although there was no effect of emotional experience on categorization latencies. These results will be examined in more detail in the Discussion section.

### Abstract SCT

The hierarchical multiple regression results are shown in Table 6. There are again two important results that should be highlighted. First, at step 1 of the analyses, concreteness had significant positive semi-partial correlations for both categorization latencies and categorization errors. That is, concreteness exerted a facilitatory effect, such that lower concreteness ratings (i.e., higher typicality ratings for the “abstract” category, see footnote 2) were associated with faster and more accurate categorizations (which is exactly what would be expected from “abstract” typicality ratings). Once more, the within-category variability accounted for by concreteness (or typicality) allowed for a more stringent test of the effects of the three dimensions of experiential knowledge at step 2 of the analyses. Second, and importantly, as can be seen in Table 6, for both criterion variables, there was a significant change in *R*^2^ when the three dimensions of experiential knowledge were added to the analyses.

**Table 6:** **Results of hierarchical multiple regression analyses for the abstract SCT**.

Variable	*B*	*SEB*	β	*sr*	Δ*R^2^*	*R^2^*
**CATEGORIZATION LATENCY**						
Step 1						0.22***
Freq	−0.01	0.02	−0.06	−0.03		
AoA	0.01	0.01	0.05	0.04		
LOD	−0.05	0.04	−0.12	−0.08		
Phonemes	0.04	0.02	0.23	0.13		
Syllables	0.05	0.04	0.15	0.09		
Morphemes	−0.05	0.04	−0.11	−0.08		
Concreteness	0.12	0.03	0.31	0.30***		
Senses	−0.01	0.01	−0.08	−0.07		
NCOUNT−INV	0.11	0.06	0.16	0.11		
Step 2					0.15***	0.37***
EE	−0.11	0.02	−0.45	−0.35***		
Imageability	0.00	0.04	−0.01	−0.01		
BOI	0.16	0.06	0.19	0.16**		
**CATEGORIZATION ERROR**						
Step 1						0.17***
Freq	0.18	0.38	0.06	0.03		
AoA	0.00	0.24	0.00	0.00		
LOD	−0.55	0.94	−0.06	−0.04		
Phonemes	0.05	0.44	0.02	0.01		
Syllables	0.17	0.82	0.02	0.01		
Morphemes	0.31	0.81	0.04	0.03		
Concreteness	3.43	0.61	0.40	0.38***		
Senses	−0.37	0.29	−0.10	−0.09		
NCOUNT−INV	1.14	1.44	0.08	0.05		
Step 2					0.13***	0.30***
EE	−1.79	0.45	−0.33	−0.25***		
Imageability	−0.14	0.85	−0.01	−0.01		
BOI	6.11	1.35	0.34	0.29***		

*p < 0.05, **p < 0.01, ***p < 0.001.

*Freq, HAL log-frequency; AoA, age of acquisition; LOD, Levenshtein orthographic distance; NCOUNT-INV, inverse of number of word neighbors plus 1; EE, emotional experience; BOI, body-object interaction*.

Recall that according to Vigliocco et al.’s (2009) framework of semantic representation, emotional knowledge is diagnostic of abstract concepts. We therefore had predicted that emotional experience should exert facilitatory effects. This prediction was supported for both the categorization latency and categorization error data, such that higher emotional experience ratings were associated with faster and more accurate categorizations. Our predictions regarding the effects of imageability and BOI were more speculative. One possibility we suggested was that because these two dimensions are diagnostic of concrete concepts, they may inhibit categorization, because they are not congruent with the decision criterion of “is the word abstract?”. A second possibility we suggested was that these two dimensions may exert no effects (again, for the potential reasons outlined above). The data provided mixed support for the two predictions. On the one hand, BOI exerted inhibitory effects on categorization latencies and errors, such that higher BOI ratings were associated with slower and less accurate categorizations. On the other hand, imageability exerted no effect on either categorization latencies or errors. Again, these results will be examined in more detail in the Discussion section.

## Discussion

According to the PSS framework of grounded cognition, conceptual processing involves simulation, or the partial reenactment of the neural states involved during bodily interaction with the environment (Barsalou, 1999). More recently, Barsalou (2003, 2008, 2009) elaborated PSS to include the idea of situated conceptualization: representations underlying conceptual knowledge include much of the rich information associated with the environmental contexts in which those concepts were acquired. Thus, simulation of conceptual knowledge involves many forms of neural reenactment, such as sensory, motor, and emotional neural reenactment (Wilson-Mendenhall et al., 2011).

As mentioned, the PSS framework has previously been used to explain the effects of imageability and BOI in lexical processing. In conjunction with PSS, the semantic feedback activation framework has been used to provide a specific account for how effects of imageability and BOI arise within the visual word recognition system. The basic idea is that easily imageable words and high BOI words are semantically richer, and thus they generate greater amounts of semantic activation (i.e., richer sensory simulations and richer motor simulations) within semantic units. Facilitatory effects of imageability and BOI are observed in such tasks as lexical decision and word naming because the greater amount of semantic activation generated by easily imageable words and high BOI words leads to greater semantic feedback to orthographic units and to phonological units, which leads to faster settling of orthographic representations and phonological representations, respectively. Facilitatory effects of the above dimensions are observed in semantic categorization because the greater amount of semantic activation generated by easily imageable words and high BOI words leads to faster settling of semantic representations.

An important consideration when examining the effects of a particular semantic dimension, particularly for the present study in which the SCT was used, is that given the dynamic nature of semantic processing (e.g., Kiefer and Pulvermüller, 2012), the effect of any particular dimension is likely to be a function of both bottom-up processing (e.g., semantically richer words elicit greater levels of semantic activation) and the top-down influence of task demands. For example, the vast majority of studies examining the effects of BOI in the SCT have used imageability (e.g., Wellsby et al., 2011) or concreteness (Bennett et al., 2011) decision criteria. All these studies reported facilitatory effects of BOI. The explanation offered is that the increased semantic activation (or richer motor simulations) elicited by high BOI words provided evidence consistent with the demands of the task (e.g., respond only if the word is easily imageable or is concrete). However, Tousignant and Pexman (2012) explicitly manipulated the instructions given to their participants. More specifically, in three of their SCTs, participants knew that “entity” (concrete thing) was part of the decision category. In their first SCT, participants were instructed to press one button for words referring to entities and another button for words referring to non-entities. In their second and third SCTs, participants were told to press one button for words referring to entities and another for words referring to actions (the order of presentation in the instructions of which buttons to press for entity and action words were reversed in these two SCTs). In a fourth SCT, participants were instructed to press one button for words referring to actions and another for words referring to non-actions. Thus, in the fourth SCT, there was no explicit mention of entities in the instructions. Tousignant and Pexman (2012) reported facilitatory effects of BOI only for the three SCTs in which “entity” explicitly comprised part of the decision category. For these three SCTs, the increased semantic activation (or richer motor simulations) elicited by high BOI words provided evidence consistent with the demands of the task (e.g., respond in a specific way to words referring to entities). For the fourth SCT, the increased semantic activation (or richer motor simulations) elicited by high BOI words did not provide evidence consistent with the demands of the task, as it was essentially an “action versus no action” decision category, and thus no effect of BOI was observed. This consideration of the interaction between semantic activation and task demands will be important in our discussion below of the nature of the effects observed in the present study.

As noted, the purpose of the present study was to examine the effects of emotional experience, imageability, and BOI in semantic processing for concrete and abstract nouns. According to Vigliocco et al.’s (2009) framework of semantic representation, sensorimotor knowledge should underlie the meanings of concrete concepts, whereas emotional knowledge should underlie the meanings of abstract concepts. Based on this framework, we made two general sets of predictions, which we will address in turn.

First, we predicted that when any dimension of experiential knowledge is diagnostic of a type of concept examined in a particular SCT, the knowledge that dimension brings to bear should be congruent with the decision criterion of that SCT, and should thus lead to facilitation of task performance. What this means for the present study is that in the concrete SCT, because imageability and BOI are diagnostic of concrete concepts, higher ratings on these two dimensions should lead to faster and more accurate concreteness categorizations, whereas in the abstract SCT, because emotional experience is diagnostic of abstract concepts, higher ratings on this dimension should lead to faster and more accurate abstractness categorizations. All these predictions were supported, such that there were facilitatory effects of imageability and BOI in the concrete SCT (i.e., higher ratings on these two dimensions were associated with faster and more accurate concreteness categorizations), and there were facilitatory effects of emotional experience in the abstract SCT (i.e., higher ratings on this dimension were associated with faster and more accurate abstractness categorizations).

These results regarding the facilitatory effects of imageability and BOI in the concrete SCT and of emotional experience in the abstract SCT provide one important source of support for the idea that imageability and BOI are diagnostic of concrete concepts and that emotional experience is diagnostic of abstract concepts (Vigliocco et al., 2009). These results also strongly support the idea, derived from the semantic feedback activation framework of visual word recognition (Hino and Lupker, 1996; Pexman et al., 2002), that when a particular dimension of experiential knowledge is congruent with task demands, task performance is facilitated. In other words, these results are consistent with the literature outlined in the Introduction demonstrating that when a semantic richness variable provides evidence consistent with task demands, semantic categorization performance is facilitated. However, an important and novel aspect of the present study is that we observed facilitatory effects of a dimension of emotional experiential knowledge (i.e., the dimension of emotional experience) in the processing of nouns referring to abstract concepts.

Second, we predicted that two possible outcomes could occur when any dimension of experiential knowledge is not diagnostic of a type of concept examined in a particular SCT. One possible outcome was that inhibitory effects would be observed, and an alternative outcome was that no effects would be observed, under these experimental conditions. For the present study, this meant that either inhibitory or null effects of imageability and BOI were expected for the abstract SCT, whereas inhibitory or null effects of emotional experience were expected for the concrete SCT. The results did not provide unequivocal support for either prediction. In the concrete SCT, there was an inhibitory effect of emotional experience on categorization errors (higher ratings of emotional experience were associated with less accurate concreteness categorizations), but there was no effect on categorization latencies. In the abstract SCT, there were inhibitory effects of BOI on both categorization latencies and errors (higher ratings of BOI were associated with slower and less accurate abstractness categorizations), but there were no effects of imageability.

These inhibitory effects of BOI in the abstract SCT and of emotional experience in the concrete SCT provide a second source of support for the idea that motor knowledge is diagnostic of concrete concepts and that emotional knowledge is diagnostic of abstract concepts (Vigliocco et al., 2009). The reason for this is that when a knowledge type is not congruent with task demands (e.g., motor knowledge is not congruent with making abstractness categorizations), the increased levels of semantic richness (e.g., richer motor simulations) do not facilitate performance (e.g., making abstractness categorizations). This provides some support for the idea that simulation is an obligatory cognitive process, and is not simply used when it may facilitate performance (e.g., using motor simulations in the concrete SCT). However, we emphasize that these findings and conclusions are tentative because the results are novel, and future research will need to be undertaken to determine whether they are reliable (i.e., can be replicated), or are due to some theoretically uninteresting reason specific to the present study (e.g., the particular stimulus sets used).

In the present results, the concreteness (or, typicality) dimension was related to the processing of concrete nouns and abstract nouns. As noted in footnote 2, the higher end of the concreteness ratings (in the Friendly et al., 1982, and Paivio et al., 1968, norms) can be treated as measuring more typical instances of the category “concrete things,” whereas the lower end of the concreteness ratings can be treated as measuring more typical instances of the category “abstract things.” In the concrete SCT, there were significant negative semi-partial correlations between concreteness and categorization latency and categorization error. These findings indicate that higher concreteness ratings (i.e., higher typicality ratings of “concrete things”) were associated with faster and more accurate categorizations. In the abstract SCT, there were significant positive semi-partial correlations between concreteness and categorization latency and categorization error. In this case, these findings indicate that lower concreteness ratings (i.e., higher typicality ratings of “abstract things”) were associated with faster and more accurate categorizations. Thus, in both SCTs, categorization was facilitated for concepts rated to be more typical of the particular category (higher concreteness ratings for the concrete SCT, but lower concreteness ratings for the abstract SCT). Including concreteness in the analyses was important, because its inclusion allowed for more stringent tests of the effects of the three dimensions of experiential knowledge; any overlapping variability shared by these dimensions with concreteness was credited to concreteness at the first step of the analyses. Hence, any variability accounted for by emotional experience, imageability, and BOI in the present study is unique and not shared with typicality or any other of the measures included in the analyses.

An interesting question that the present study cannot address, due to the go/no-go nature of the two SCTs, is what would occur if a yes/no design were used (i.e., overt button responses are made to both items requiring “yes” responses and to items requiring “no” responses). More specifically, what would be the effects of emotional experience for abstract nouns presented on “no” trials in a concrete SCT, and what would be the effects of imageability and BOI for concrete nouns presented on “no” trials in an abstract SCT? Based on the semantic feedback activation framework of visual word recognition, the following predictions can be made. First, in the concrete SCT, because emotional experience is diagnostic of abstract concepts, lower ratings on this dimension would be associated with the noun being considered less abstract, or in other words, being considered more concrete, which would likely lead to inhibitory effects because these nouns would be more difficult to differentiate from concrete nouns. Second, in the abstract SCT, because imageability and BOI are diagnostic of concrete concepts, lower ratings on these two dimensions would be associated with the noun being considered less concrete, or in other words, being considered more abstract, which would likely lead to inhibitory effects because these nouns would be more difficult to differentiate from abstract nouns. Of course, these predictions must await testing in future research.

A final and important issue that was not directly addressed in the present study was how the dimension of emotional experience may be related to other dimensions of emotionality, such as valence and arousal, that have been used in the literature to assess the influence of emotional knowledge in lexical processing. Kousta et al. (2009, 2011) have demonstrated that valence and arousal significantly influence lexical processing in the lexical decision task. It is therefore important to examine how different measures of emotional experiential knowledge are related and of their effects in lexical processing.

To examine the specific issues of the relationships between the dimensions of emotional experience, valence, and arousal, and their effects on categorization latency and errors in the present study, we did the following. First, we obtained valence and arousal values from the Affective Norms for English Words (ANEW) database (Bradley and Lang, 1999), which were available for 87 of the concrete nouns and 69 of the abstract nouns. Second, we conducted separate *post hoc* simultaneous multiple regression analyses for each data set. (We conducted simultaneous regression analyses rather than hierarchical regression analyses because of the reduction of statistical power due to the smaller number of stimuli in each analysis.) All the lexical and semantic variables that were entered in the follow up analyses above were entered in the *post hoc* analyses, along with valence and arousal. We emphasize that these analyses are exploratory in nature, and any conclusions that may be derived from them are tentative and must await further experimentation.

For the concrete nouns, the zero-order correlations between valence and arousal, valence and emotional experience, and arousal and emotional experience were *r*(87) = 0.34 (*p* < 0.01), 0.41 (*p* < 0.001), and 0.57 (*p* < 0.001), respectively. For the abstract nouns, the zero-order correlations between valence and arousal, valence and emotional experience, and arousal and emotional experience were *r*(69) = 0.11 (*ns*), −0.08 (*ns*), and 0.60 (*p* < 0.001), respectively. These correlations suggest that the dimension of emotional experience is positively related to the dimension of arousal for both concrete nouns and abstract nouns, whereas it is only positively related to the dimension of valence for the concrete nouns. The positive relationship between emotional experience and valence is perhaps not surprising, considering that valence was an emotional characteristic that is salient in the instructions used to obtain the emotional experience ratings.

For the concrete SCT regression analyses, although none of the three dimensions of emotional experiential knowledge were significantly related to categorization latencies, valence was significantly related to categorization errors, such that higher ratings of valence were associated with less accurate categorization (*sr* = 0.15). For the abstract SCT regression analyses, only emotional experience was significantly related to categorization latency, such that higher ratings of emotional experience were associated with faster latencies (*sr* = −0.39), and none of the dimensions of emotional experiential knowledge were significantly related to categorization errors. The most important finding from the *post hoc* regression analyses was that although emotional experience and arousal were significantly positively correlated for the abstract noun stimulus set, emotional experience continued to exert a facilitatory effect on categorization latencies in the abstract SCT, even with arousal in the analysis. This finding provides further support for the idea that the dimension of emotional experience is a robust measure of emotional experiential knowledge. Of course, further research is needed to determine how this dimension is related to other dimensions of emotional experiential knowledge, and of their influence in visual word recognition tasks other than semantic categorization.

## Conflict of Interest Statement

The authors declare that the research was conducted in the absence of any commercial or financial relationships that could be construed as a potential conflict of interest.

## Footnotes

^1^See Barsalou et al. (2008) for a related framework they call Language and Situated Simulation (LASS).

^2^Several studies have demonstrated the importance of typicality in semantic categorization. For example, Hampton (1997) reported that typicality accounted for significant amounts of unique within-category variability in both categorization latency and response probability across a variety of categories (see also, Casey, 1992; Larochelle and Pineau, 1994; and Smith et al., 1974, for similar results). Because the two categories used in the present study were quite broad, namely whether nouns were concrete or abstract, we used the concreteness ratings from the Friendly et al. (1982) and the Paivio et al. (1968) norms as typicality ratings. The instructions used in these studies included the following, “Each word was accompanied by a seven-point bipolar numerical scale, with the extremes labeled Highly Abstract and Highly Concrete, respectively…the ends of the scale were defined in terms of abstractness-concreteness rather than low concreteness-high concreteness…the present instructions stated that, ‘Any word that refers to objects, materials, or persons should receive a *high concreteness* rating; any word that refers to an abstract concept that cannot be experienced by the senses should receive a *high abstractness* rating”’ (Paivio et al., 1968, p. 5). Thus, the lower ratings associated with the abstract words provided an indication of their relative “abstractness” (as was indicated at the lower end of the scale), and are therefore appropriate for entry into the analysis of the abstract categorization task data. The higher ratings associated with the concrete words provided an indication of their relative “concreteness” (as was indicated at the higher end of the scale), and are therefore appropriate for entry into the analysis of the concrete categorization task data. Inclusion of these ratings is important, because they will account for within-category variability, and therefore allow for a more stringent test of whether the three dimensions of experiential knowledge account for additional categorization latency and error variability, above and beyond that accounted for by the concreteness ratings (and the other variables entered in the first step of the analyses).

^3^Shaoul and Westbury (2010a) developed the High Dimensional Explorer (HiDEx) model of lexical co-occurrence, based on the basic architecture of the HAL model (Lund and Burgess, 1996). One of the measures that HiDEx computes is called NCOUNT-INV. This measure is the inverse (plus 1) of another measure HiDEx computes, called NCOUNT, which is the number of neighbor words within a specified neighborhood membership threshold. NCOUNT-INV “has a value of 1 for words with no neighbors and smaller values for words with more neighbors” (p. 397). It is essentially a measure of the lexical co-occurrence neighborhood size for a given word. We used the NCOUNT-INV measure because Shaoul and Westbury (2010a) reported that it best correlated with the SCT they used in their study (the decision category was whether words referred to living things).

^4^http://www.empirisoft.com/DirectRT.aspx

^5^We did not include number of letters in any of the multiple regression analyses, because of the high zero-order correlations between this variable and the Levenshtein orthographic distance and number of phonemes variables for both the concrete and the abstract noun sets.

## Appendix

### Concrete nouns used in the experiments

**Table:**

*accordion	cauliflower	*destroyer	jitterbug	person
acid	chairman	diamond	journal	picture
agent	chapter	dinner	kitchen	pitcher
alligator	cherry	*disease	lady	planet
aluminum	chestnut	dishwasher	*laughter	platform
ambassador	chickenpox	dollar	leader	player
apple	chimney	doorway	leather	poem
arrow	chinchilla	dragon	letter	police
artist	chopstick	eagle	lieutenant	pony
asparagus	cigarette	earthworm	lion	prairie
auditorium	circle	elbow	luncheon	projector
author	city	empire	machine	propeller
baby	clarinet	engine	madam	province
bacteria	*climate	envelope	marshmallow	quarter
barrel	closet	estate	mayor	railway
basement	clothing	*evening	*meeting	rattlesnake
basin	coffee	fabric	member	rectangle
bedroom	collar	farmer	merchant	refrigerator
berry	college	finger	metal	sandwich
binoculars	colonel	flashbulb	mirror	scholar
blacksmith	color	football	mistress	screwdriver
blanket	column	forehead	*moisture	secretary
body	compass	forest	monarch	sentence
building	comrade	fountain	monastery	servant
bullet	concert	freckles	money	shepherd
*business	contract	garden	monster	sheriff
butcher	corner	grasshopper	mother	sparrow
butter	costume	handlebars	motor	speaker
cabin	cotton	headboard	mountain	squirrel
canal	country	helmet	mouthpiece	station
candy	couple	highway	navy	steamer
cannon	cousin	hotel	number	stomach
canoe	creature	hunter	orchard	student
captain	crocodile	hurricane	oven	summer
cardinal	crystal	husband	painter	thermometer
carpet	cupboards	*illness	painting	trapezoid
carriage	dandruff	island	partner	treasurer
castle	daughter	jacket	pasture	tuberculosis
caterpillar	daylight	jellyfish	penicillin	window
cattle	dealer	jewel	perfume	winter

### Abstract nouns used in the experiments

**Table:**

aberration	contrast	fallacy	*jeopardy	prestige
ability	control	fantasy	judgment	quality
absence	courage	fate	*justice	rating
accord	crisis	favor	knowledge	reaction
*account	criterion	feature	legend	reason
advance	custom	feeling	limit	reform
adversity	danger	feint	maker	regard
advice	deceit	feudalism	malice	relief
afterlife	decline	figment	manner	request
agreement	decrease	folly	marvel	reserve
allegory	deduction	forethought	mastery	revenge
amount	degree	fortune	meaning	review
appeal	delay	freedom	meantime	satire
approach	democracy	future	memory	sensation
aptitude	desire	gist	menace	*sister
array	devotion	gratitude	mercy	situation
aspect	*dijon	greed	merit	sobriety
atrocity	discipline	habit	method	soul
attempt	disclosure	heredity	mind	spirit
attitude	discretion	hindrance	miracle	status
attribute	disposition	*honor	*moment	support
banality	distraction	hope	mood	suppression
basis	distress	*hour	necessity	suspect
belief	duty	hypothesis	neglect	temerity
betrayal	eccentricity	idea	non-sense	tendency
blandness	economy	ignorance	nothing	theory
boredom	effect	illusion	notion	tribute
capacity	effort	*image	obedience	*trifle
chance	emancipation	immunity	obsession	trouble
clemency	envy	impulse	offense	truth
comment	equity	inanity	offer	unification
comparison	error	incident	opinion	upkeep
competence	essence	incline	opportunity	value
*compound	exclusion	inducement	outcome	vanity
concept	export	ingratitude	pacifism	venture
concern	expression	instance	pardon	violation
confidence	extent	instant	patience	virtue
conflict	*facility	intellect	perception	weakness
consent	factor	interest	perjury	welfare
context	failure	irony	pledge	wonder

### Written instructions used for the emotional experience rating task

Words differ in the extent to which they elicit or evoke an emotional experience. Some words elicit or evoke strong emotional experiences (e.g., JUSTICE), whereas other words elicit or evoke weaker emotional experiences (e.g., MOMENT). The purpose of this experiment is to rate words as to the ease with which they elicit or evoke emotional experience. For example, the word “justice” refers to a concept that is associated with high levels of emotional experience (e.g., think of the emotional conditions that arise when a jury verdict is delivered, such as joy, dismay, anger, frustration), whereas the word “moment” refers to a concept that is associated with low levels of emotional experience (i.e., it is difficult to think of any kind of emotional experience to which this word is related). Any word (e.g., “justice”) that in your estimation elicits or evokes high levels of emotional experience should be given a high emotional experience rating (at the upper end of the numerical scale). Any word (e.g., “moment”) that in your estimation elicits or evokes low levels of emotional experience should be given a low emotional experience rating (at the lower end of the scale). Because words tend to make you think of other words as associates, it is important that your ratings *not* be based on this and that you judge only the ease with which a word elicits or evokes emotional experience. Remember, all the words are nouns and you should base your ratings on this fact.

Your emotional experience ratings will be made on a 1–7 scale. A value of 1 will indicate a low emotional experience rating, and a value of 7 will indicate a high emotional experience rating. Values of 2–6 will indicate intermediate ratings. Please feel free to use the whole range of values provided when making your ratings. Circle the rating that is most appropriate for each word. When making your ratings, try to be as accurate as possible, but do not spend too much time on any one word.

**Table:**

1		2		3		4		5		6		7
Low						Medium						High
